# C-STrap Sample Preparation Method—*In-Situ* Cysteinyl Peptide Capture for Bottom-Up Proteomics Analysis in the STrap Format

**DOI:** 10.1371/journal.pone.0138775

**Published:** 2015-09-25

**Authors:** Alexandre Zougman, Rosamonde E. Banks

**Affiliations:** Clinical and Biomedical Proteomics Group, Cancer Research UK Centre, Leeds Institute of Cancer and Pathology, St James's University Hospital, Leeds, United Kingdom; Consejo Superior de Investigaciones Cientificas, SPAIN

## Abstract

Recently we introduced the concept of Suspension Trapping (STrap) for bottom-up proteomics sample processing that is based upon SDS-mediated protein extraction, swift detergent removal and rapid reactor-type protein digestion in a quartz depth filter trap. As the depth filter surface is made of silica, it is readily modifiable with various functional groups using the silane coupling chemistries. Thus, during the digest, peptides possessing specific features could be targeted for enrichment by the functionalized depth filter material while non-targeted peptides could be collected as an unbound distinct fraction after the digest. In the example presented here the quartz depth filter surface is functionalized with the pyridyldithiol group therefore enabling reversible *in-situ* capture of the cysteine-containing peptides generated during the STrap-based digest. The described C-STrap method retains all advantages of the original STrap methodology and provides robust foundation for the conception of the targeted *in-situ* peptide fractionation in the STrap format for bottom-up proteomics. The presented data support the method’s use in qualitative and semi-quantitative proteomics experiments.

## Introduction

Current state-of-the-art mass spectrometry (MS) instrumentation employed in proteomics handles a ‘mere’ four orders of magnitude of the protein dynamic range, compared with the no less than seven-orders-of-magnitude spanning protein abundances in living organisms [1]. Reducing the complexity of peptide mixtures is one of the straightforward means to increase the depth of coverage in shotgun proteomics experiments. One approach is by selective enrichment of the proteolytic peptides based on their chemical composition. Cysteinyl (Cys) peptides containing nucleophilic, easily oxidizable thiol groups, constitute about a quarter of the *in-silico* digested tryptic human proteome [2] and, therefore, are very practical enrichment targets in proteomics studies. Generally, two main approaches have been employed to target the cysteinyl peptides. These are based either on reversible sulfhydryl immobilization on a resin using disulfide exchange or on covalent modification by an alkylating probe carrying a tag with the consequent enrichment of the tagged peptides on an anti-tag resin (e.g. biotin-avidin reaction). Thiopropyl Sepharose™ 6B (GE Healthcare) has been used widely over several decades to reversibly bind biological molecules containing the sulfhydryl groups—the 2-thiopyridyl disulphide group of the sepharose matrix reacts with a molecule containing a free thiol to form a mixed disulfide; during the reaction thiopyridone is formed while the bound target molecule is released by addition of a reducing agent. In 1980, before the onset of the “proteomics” age, this form of covalent chromatography was used, for example, to isolate and characterize some tryptic cysteinyl peptides derived from phosphorylase enzymes [3]. Twenty five years later, the same approach was employed in a true proteomics study characterizing the mammary epithelial cell proteome by targeted enrichment of the cysteine-containing peptides [2]. The same principle was also used in the design of super-paramagnetic nanoparticles for selective cysteinyl peptides enrichment [4]. An alternative strategy, the selective isolation and labeling of the cysteinyl peptides (isotope-coded affinity tags (ICAT) method), was reported in 1999 by Gygi et al. [5] beginning a new era in the label-based quantitative proteomics. In this case, the reduced cysteine residues in peptides were covalently labeled with biotinylated isotopic tags carrying a thiol-reactive iodoacetamide group before being captured on avidin columns and recovered subsequently by acidic elution. Cleavable ICAT linkers were subsequently introduced to improve the method’s performance [6]. In addition to the above approaches the enrichment of the thiol-containing peptides using a zinc(II)–cyclen functionality has been reported [7].

Recently, we introduced the Suspension Trapping (STrap) sample preparation method for simple, rapid and unbiased bottom-up processing of proteomics samples [8]. The basic principle underlying the STrap methodology is creation of fine particles in suspension from SDS-solubilized proteins, capture of the particles in a quartz depth filter with subsequent *in-situ* digestion by an introduced protease. Originally, the quartz depth filters were chosen because of their low peptide background binding under the digest conditions, as well as commercial availability. We later hypothesized that since the depth filter surface is made of silica, it presents a unique opportunity for functionalization with silane coupling chemistries. Thus, during the digestion process, the peptides possessing the targeted functionalities are selectively enriched by the modified depth filter material. Importantly, the reported C-STrap approach retains the above-mentioned key advantages of the STrap processing as such.

## Materials and Methods

### The C-STrap tip design

The MK360 Munktell quartz filter is modified with pyridyldithiol, i.e. a MK360-50 mm filter disk is incubated with 10 ml of 2% (3-Aminopropyl)trimethoxysilane (APTMS) solution in acetone for 2 hours and then washed several times with acetone. 7 mg of *N*-succinimidyl 3-(2-pyridyldithiol) propionate (SPDP) is dissolved in 0.5 ml of dimethyl sulfoxide (DMSO) and added into 9.5 ml of the phosphate buffered saline (PBS)/EDTA solution (137 mM NaCl, 2.7 mM KCl, 10 mM Na_2_HPO_4_, 1.8 mM KH_2_PO_4_, pH 7.4/15 mM EDTA). Next, the aminopropyl-modified filter is incubated with the resultant SPDP solution for 6 hours (or overnight) at room temperature (RT), and then washed several times with the PBS/EDTA solution and air dried. Using a 14 gauge blunt needle, the basic C-STrap tip is constructed by inserting 12 plugs of the pyridyldithiol modified MK360 filter and 3 plugs of the Empore C_18_ extraction disk material into a 200 μl pipette tip—similarly to the original STrap protocol [8]. For the alternative approach, the only quartz (OQ) C-STrap tip is constructed by inserting 12 plugs of the pyridyldithiol modified MK360 material into a 200 μl pipette tip—no underlying C_18_ membrane compartment is added in this case.

### HeLa lysate processing

A HeLa cell pellet of 8 million cells (50 μl of volume) was lysed in excess lysis solution (750 μl of 5% SDS in 50 mM TRIS-HCl, pH 7.6) at RT. To shear the DNA the sample was sonicated briefly with a probe sonicator. Dithiothreitol was then added to a final concentration of 20 mM. The extract was heated at 95°C for 5 min and then clarified by centrifugation at 12,100 x g for 10 min. Protein concentration was measured by tryptophan fluorescence [9]. 50 μg of protein was processed, in triplicate, either by the basic or OQ C-STrap protocols (S1 Text).

### mTOR immunoprecipitation

Immunoprecipitation (IP) using polyclonal goat anti-mTOR (sc-1549, Santa Cruz) and control normal goat IgG (sc-2028, Santa Cruz) antibodies was performed as described [8] from HeLa cells using 3 μg of the antibody per IP, in duplicate. The bound proteins were eluted by incubating the magnetic beads with 30 μl of 5% SDS/50 mM TRIS-HCl, pH 7.6 buffer containing 20 mM DTT at 90°C for 5 min. The eluted material was further processed by the OQ C-STrap method.

### Mass spectrometry and data analysis

Peptides were separated online by reversed-phase capillary liquid chromatography using EASY-nLC 1000 UHPLC system (Proxeon) connected to a 30-cm capillary emitter column made in-house (inner diameter 75 μm, packed with 3 μm Pursuit C_18_ media). The chromatography system was hyphenated with a linear quadrupole ion trap—orbitrap (LTQ-Orbitrap) Velos mass spectrometer (Thermo). The acquisition time per run used to analyze the peptide fractions was for the HeLa lysate processing 150 min and for the IP processing 100 min. Survey MS scans (range of 305–1350 amu) were acquired in the orbitrap with the resolution set to 60,000. Up to 20 most intense ions per scan were fragmented and analyzed in the linear trap. Data were processed against the Uniprot human protein sequence database (January, 2014) with MaxQuant 1.3.0.5 software (www.maxquant.org) [10]. The mass tolerance for MS scan was set to 7 ppm, the fragment mass tolerance for MS/MS was set to 0.5 Da. Protein N-terminal acetylation and oxidation of methionine as well as carbamidomethylation of cysteine were set as variable modifications. A maximum of two missed cleavages and at least one unique peptide for valid protein identification were chosen. The maximum protein and peptide false discovery rates were set to 0.01.

## Results and Discussion

**Fig 1:**
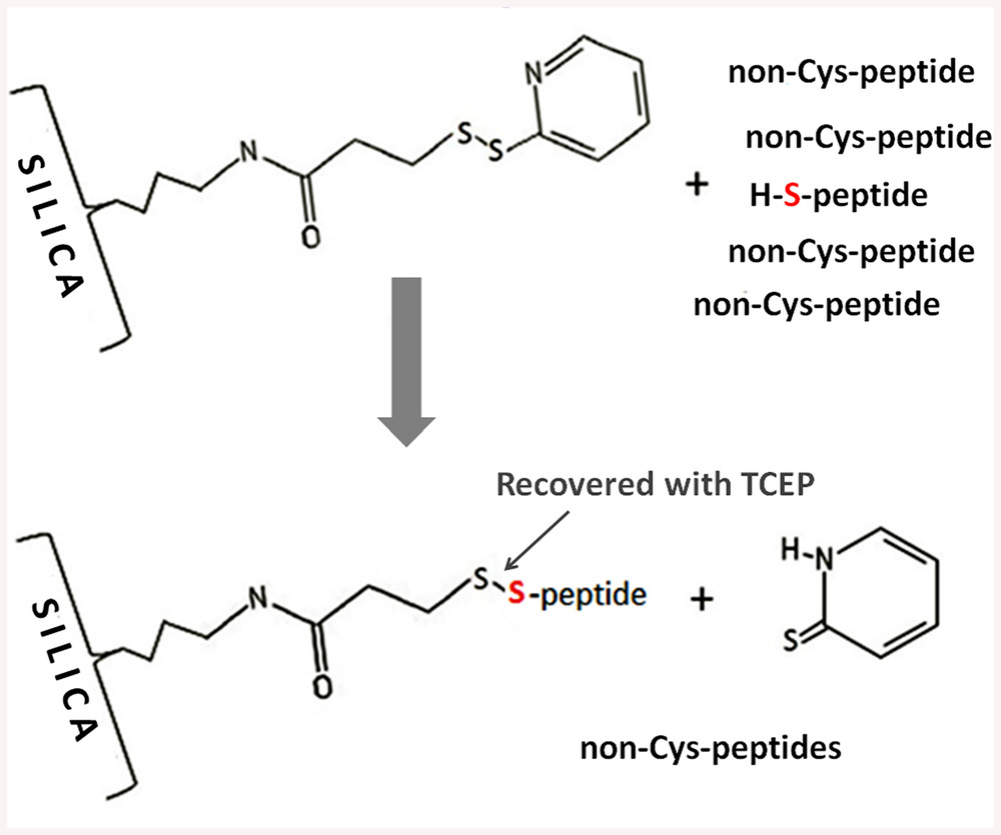
Schematic of the reversible cysteinyl peptide capture on the quartz surface functionalized with the pyridyldithiol group. The covalently bound cysteinyl-peptides are recovered with the use of a reducing agent.

To explore the proposed scheme, we activated the quartz depth filter surface with the pyridyldithiol group therefore enabling reversible *in-situ* capture of the cysteine-containing peptides (Fig 1) generated during the STrap-based digest by disulfide exchange. After the uncaptured non-cysteine containing peptides have been collected, the reversibly bound cysteinyl-peptides are recovered as a distinct fraction through the use of a reducing agent.

**Fig 2:**
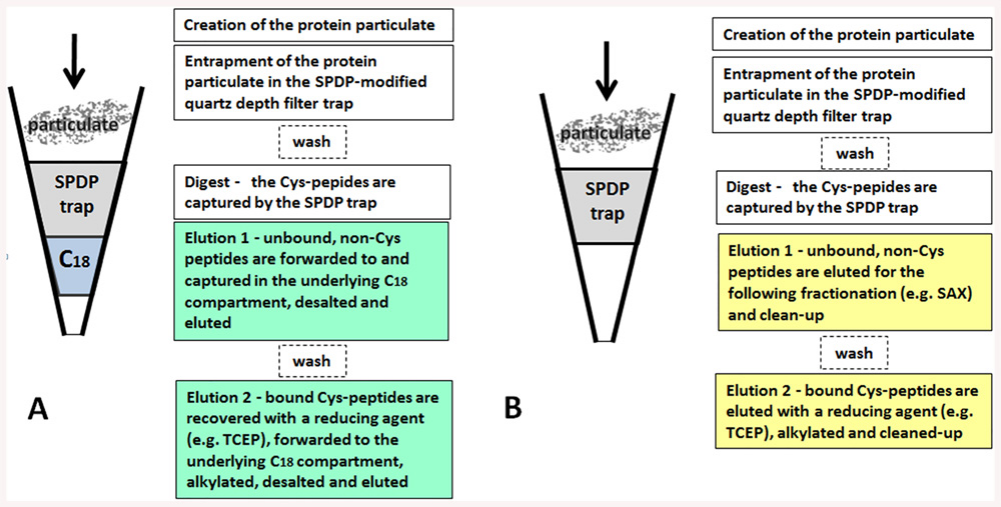
Outline of the basic (A) and only quartz (OQ) (B) C-STrap processing approaches. During the basic C-STrap procedure **(A)**, the trapped protein particulate is digested *in-situ* and the cysteinyl peptides are bound to the filter matrix. After the digest, the unbound non-Cys peptides are desalted in the underlying C_18_ compartment and eluted. The bound Cys-peptides are then recovered with a reducing agent and processed in the same C_18_ compartment. During the OQ C-STrap procedure **(B)** the *ex-situ* post-digest processing is performed—the unbound non-Cys peptides are eluted for further fractionation by strong anion exchange (SAX) into several fractions. The bound Cys-peptides are then eluted with a reducing agent and processed *ex-situ* on the C_18_ STAGE tip.

The implementation of the C-STrap technique is proposed in two ways. The first, simple approach employs the standard STrap-like design of the processing unit composed of the upper trapping compartment made of the succinimidyl 3-(2-pyridyldithio)propionate (SPDP)–functionalized quartz depth filter and underlying C_18_ part (Fig 2A). This design allows for the *in situ* protein processing and clean-up of the peptide products into two distinct, Cys and non-Cys, fractions. The SDS-solubilized reduced protein sample is introduced into the C-STrap tip, in the same way as in the original STrap method, and the particulate is created and trapped in the SPDP-quartz depth filter. An enzyme such as trypsin is introduced and the Cys-containing peptides produced during the digest are covalently bound to the depth filter matrix. After digestion, the unbound non-Cys peptides are moved to the C_18_ compartment, desalted and eluted. The bound Cys-peptides are then recovered with a reducing agent, moved to the C_18_ compartment, alkylated *in situ*, desalted and eluted. Alternatively, the second more laborious approach results in additional peptide fractions and therefore could be used when deeper peptide coverage is required. This approach employs the only quartz (OQ) C-STrap tip containing just the SPDP-modified quartz compartment without the underlying C_18_ component which provides the advantage of reduced backpressure but involves *ex-situ* post-digest processing (Fig 2B). As in the first method, the SDS-solubilized reduced protein sample is introduced into the OQ C-STrap tip, the digest is performed and the Cys-peptides are bound to the filter matrix. Subsequently, unbound non-Cys peptides are eluted, further fractionated by strong anion exchange (SAX) into several fractions and cleaned-up. The bound Cys-peptides are then eluted with a reducing agent, applied to the C_18_ STAGE tip [11], alkylated *in-situ*, and cleaned-up.

**Fig 3:**
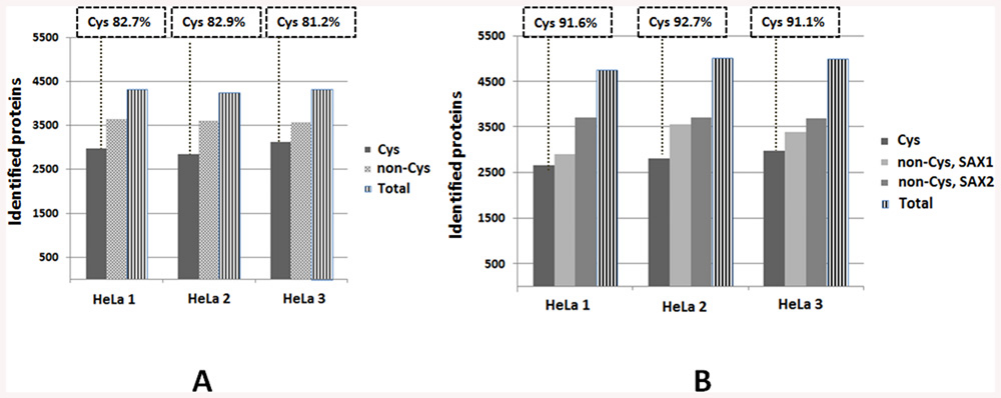
C-STrap processing of 50 μg of protein from HeLa lysates from each of 3 replicates, using either the basic (A) or only quartz (OQ) (B) approaches, followed by the LC-MS/MS analysis. On average per sample we identified 4280 proteins with the basic approach and 4930 proteins with the OQ approach. The occurrence of the cysteinyl peptides in the Cys fractions was 82.3% and 91.8% (average per sample) using the basic and OQ approaches, respectively.

To illustrate the applicability of these approaches towards processing complex proteomes, 50 μg of protein from a HeLa cell lysate was digested using trypsin, in triplicate. The lysates were processed with either the C-STrap (2 final fractions—Cys and non-Cys peptides) or OQ C-STrap (3 final fractions—Cys and two SAX non-Cys fractions) methods using 150-min LC-MS/MS runs on LTQ Orbitrap Velos mass spectrometer. An average of 4280 proteins/sample was identified with the basic C-STrap approach and 4930 proteins with the OQ C-STrap approach (Fig 3A and 3B). With the more minimal basic approach, the Cys fractions contained an average of 82.3% cysteine peptides, whereas using the OQ approach increased this proportion to 91.8%, implying some proneness of the basic methodology to carryover of the non-Cys peptides, most likely due to the fact that the clean-up of both non-Cys and Cys peptide fractions is done *in-situ* in the same C_18_ compartment.

**Fig 4:**
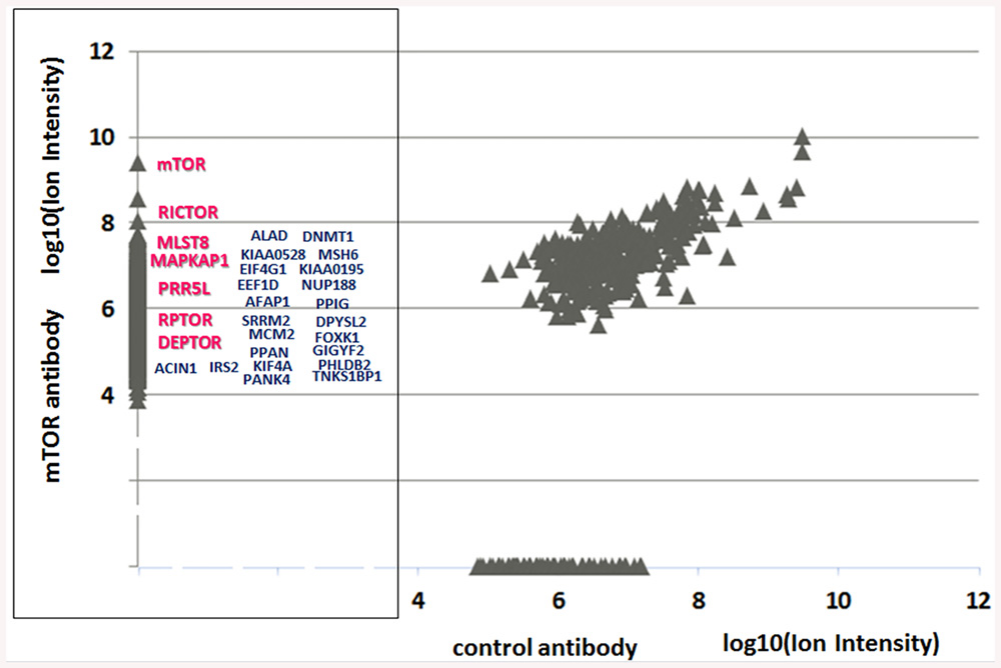
C-STrap processing of the mTOR co-immunoprecipitated proteins by the OQ approach followed by the LC-MS/MS analysis. In addition to the conventional members of the mTORC1 (RAPTOR, DEPTOR, and MLST8) and mTORC2 (RICTOR, MAPKAP1, PRR5L, and DEPTOR) complexes (shown in red), we as well were able to pinpoint some previously suggested mTOR-targeted phosphorylation substrates (shown in blue) such as KIAA0528, for example, thus providing evidence in support of the direct interaction of these proteins with the mTOR complexes.

The method can also be used to rapidly analyze less complex protein mixtures such as immunoprecipitates, for example. In order to illustrate this, immunoprecipitates (in duplicates) of the mammalian target of rapamycin (mTOR) protein, one of the hub molecules regulating cellular growth and metabolism, were prepared using 3 μg of the well-described anti-mTOR N-19 goat polyclonal antibody [12] and control goat antibody, and the OQ C-STrap processing. In the pool of co-precipitated proteins, in addition to the established key mTOR interactors, the members of the mTORC1 and mTORC2 complexes, proteins previously reported as the mTOR-targeted substrates in the quantitative mTOR-regulated phosphoproteome study were identified [13], thus providing evidence in support of the direct interaction of these putative targets with the mTOR complexes (Fig 4). The presence in this pool of such proteins as KIAA0528 (CDP138) [14], for example, supports a direct link of the mTOR hub with the insulin-stimulated glucose membrane transport machinery.

The described C-STrap technique provides the resource both for rapid digest of the SDS-solubilized proteins and consequent peptide fractionation based on the reversible capture of the cysteine-containing peptides, retaining all benefits of the original STrap methodology. The presented data support the method’s use in qualitative and semi-quantitative proteomics experiments. It is our belief that the proposed *in-situ* peptide enrichment conception for the STrap technology is not limited to the covalent capture of the cysteinyl peptides only and, in the OQ format, could potentially be used with other molecular probes targeting either specific amino acids or amino acid modifications as long as the digest conditions do not interfere with the target-probe interactions—e.g. the quartz depth filter could be derivatized with (3 Glycidyloxypropyl) trimethoxysilane (GLYMO) and then NH2-modified aptamer probes could be covalently attached to the silica surface (e.g. such as the aptamers against L-arginine to enrich for arginine-containing peptides [15]) or, by the same token, the filter may possibly be functionalized with aminophenylboronic acid to enrich for glycopeptides [16].

## Supporting Information

S1 Text Supporting C-STrap protocols. (PDF)
