# Supplementary material for: C-STrap Sample Preparation Method—In-Situ Cysteinyl Peptide Capture for Bottom-Up Proteomics Analysis in the STrap Format
Source: PLoS One. 2015 Sep 25;10(9):e0138775. doi: 10.1371/journal.pone.0138775 (PMC4583295; doi:10.1371/journal.pone.0138775)
Supplement: S1 Text — (PDF) [file pone.0138775.s001.pdf]

Basic (simple) C-STrap method

The C-STrap tip contains the SPDP-modified depth filter compartment and the underlying C_{18} part – the peptide processing and clean-up is performed *in situ*.
Tryptic digest with enrichment of the cysteine-containing peptides, maximum protein load 50 µg. The method produces 2 peptide fractions - non-Cys and Cys peptides.

1. Materials

Solutions and Reagents

Milli-Q water (H_2O)

AmBic (Ammonium Bicarbonate) solution: 40 mM NH_4HCO_3 in H_2O

ACN solution: 50% acetonitrile in 0.5% formic acid

Lysis buffer: 5% (w/v) sodium dodecyl sulfate (SDS), 50 mM Tris/HCl pH 7.6

STrapping buffer: 90% methanol, 100 mM Tris/HCl pH 7.1

Phosphoric acid solution: 12.15% in H_2O

DTT solution: 1 M dithiothreitol in H_2O, freshly prepared

TCEP elution solution: 40 mM tris(2-carboxyethyl)phosphine in H_2O, freshly prepared

IAA solution: 40 mM iodoacetamide in AmBic, freshly prepared

Trypsin solution: 0.07 µg/µl of trypsin (V5111, Promega) in AmBic, prepared prior to starting the digest and kept on ice

FA solution: 0.5% formic acid in H_2O

Methanol

Equipment

Bench-top centrifuge (for example MiniSpin, Eppendorf)

Probe sonicator (for example Soniprep 150, MSE)

Heating block suitable for handling microtubes (for example PHMT, Grant Bio)

Plastic syringe, 20 ml (for example 301031, BD) with a custom adapter to fit into 200 µl pipette tips

Vacuum concentrator (for example SpeedVac, Thermo)

C-STrap tip

MK360 quartz filter is modified with pyridyldithiol, i.e. MK360-50 mm filter is incubated with 10 ml of 2% (3-Aminopropyl)trimethoxysilane (APTMS) soluton in acetone for 2 hours and then washed several times with acetone. 7 mg of *N*-succinimidyl 3-(2-pyridyldithiol)propionate (SPDP) is dissolved in 0.5 ml of dimethyl sulfoxide (DMSO) and added into 9.5 ml of the phosphate buffered saline (PBS)/EDTA solution (pH 7.4, 15 mM EDTA). The aminopropyl-modified filter is incubated with the resultant SPDP solution for 6 hours (or overnight) at room temperature (RT), and then washed several times with the PBS/15 mM EDTA solution and air dried.

Using a 14 gauge blunt needle, the basic C-STrap tip is constructed by inserting 12 plugs of the pyridyldithiol modified MK360 filter and 3 plugs of the Empore C_{18} extraction disk material into a 200 µl pipette tip - similarly to the original STrap tip protocol[1].

O-tube: A 1.5 mL microcentrifuge tube (72.690.001, Sarstedt) with an opening punctured in the tube's lid (alternatively, a pipette tip adapter for microcentrifuge tubes could be used).

The C-STrap tip and O-tube comprise the **Spin-unit**

Filter tips, 10 µl (TF-300-R-S, Axygen)

2. Methods

2.1 Cell lysis and reduction of cysteine residues

Cells are lysed in excess of the Lysis buffer (ca. 1:10 sample-to-Lysis buffer volume ratios) at room temperature. To shear the DNA, the lysate is briefly sonicated with a probe sonicator. DTT solution is added to the final concentration of 20 mM. The extract is heated up at 95°C for 5 min. The extract is clarified by centrifugation at ~12,000 x g for 10 min.

2.2 Preparation of the Trypsin solution

Trypsin solution is prepared prior to the step 2.3 and placed on ice.

2.3 Sample processing

1. Pre-heat the heating block to 47°C.
2. (S**ee Notes**) Add 120 µl of the STrapping buffer into the C-STrap tip onto the top of the quartz stack. Wait for 1 min.
3. (S**ee Notes**) To 18 µl of the sample add 2 µl of the Phosphoric acid stock solution. Mix by pipetting up and down.
4. Slowly add the acidified sample into the upper quarter of the STrapping buffer in the C-STrap tip. Insert the C-STrap tip into the O-tube. Place the Spin-unit into the centrifuge and mark the C-STrap tip part facing outwards.
5. Centrifuge the Spin-unit at 3000 x g for 1 min. Dispose of the tube with the flow-through.
6. Add 70 µl of the STrapping buffer into the C-STrap tip. Insert the tip into the fresh O-tube. Place the Spin-unit into the centrifuge with the C-STrap tip mark facing inwards. Centrifuge the Spin-unit for 30 sec at 3000 x g.
7. (Optional) Add 40 µl of the AmBic solution into the C-STrap tip and centrifuge the Spin-unit for 30 sec at 3000 x g. Dispose of the tube with the flow-through.
8. Add 30 µL of the Trypsin solution into the C-STrap tip onto the top of the plug stack. Push down the solution using the syringe with a customized tip adapter till the solution meniscus is positioned ca. 4 mm above the top of the plug stack.

9. Close the top of the C-STrap tip with the 10 µl filter tip.

10. Insert the closed C-STrap tip into the fresh O-tube, place the unit into the heating block at 47°C and cover with the aluminium foil. Incubate for 60 min.

11. Remove the Spin-unit from the heating device. Add 30 µl of the AmBic solution into the C-STrap tip. Centrifuge the Spin-unit at 4000 x g for 1 min.

12. Add 50 µl of the FA solution into the C-STrap tip. Centrifuge the Spin-unit at 4000 x g for 1 min. Dispose of the tube with the flow-through.

13. Elute the non-Cys peptides with 40 µl of the ACN solution into a clean sample tube. The eluted non-Cys peptides are concentrated with SpeedVac.

14. The C-STrap tip is washed twice with 70 µl of the ACN solution using centrifugation and decanting the flow-through when necessary.

15. The C-STrap tip is washed consecutively with 50 µl of methanol and 30 µl of the TCEP solution.

16. 30 µl of the TCEP elution solution is added into the C-STrap tip. The TCEP solution is pushed down with the syringe-adapter till the solution meniscus is positioned ca. 4 mm above the top of the plug stack. The tip is placed into a fresh O-tube. Incubation is performed for 30 min at room temperature.

17. 40 µl of the AmBic solution is added into the C-STrap tip. The Spin-unit is centrifuged at 4000 x g for 1 min. Repeat this step. Dispose of the tube with the flow-through.

18. Add 40 µL of the IAA alkylation solution into the tip. Push down the solution till the solution meniscus is positioned ca. 4 mm above the top of the plug stack. The tip is placed into the fresh O-tube. Leave the unit at RT in the dark for 15 min.

19. The Spin-unit is centrifuged at 4000 x g for 1 min.

20. Add 60 µL of the FA solution into the tip. The Spin-unit is centrifuged at 4000 x g for 1 min.

21. Elute the Cys peptides with 50 µl of the ACN solution into a clean sample tube. The eluted Cys peptides are concentrated in SpeedVac.

Notes:

1. After each centrifugation step make sure that all added solution has gone through the tip.
2. The typical working ratio between the STrapping buffer in the C-STrap tip and the acidified sample is 6:1. The final concentration of the phosphoric acid after addition to the sample is 1.2%.

Only Quartz (OQ) C-STrap method

The tip contains ONLY the SPDP-modified depth filter compartment, there is no underlying C_{18} membrane – the peptide clean-up is performed *ex situ*. The method results in 2 peptide fractions – non-Cys and Cys. The non-Cys fraction could be further fractionated by the ion-exchange into 2 fractions.
Tryptic digest with enrichment of the cysteine-containing peptides, maximum protein load 50 µg.

2. Materials

Solutions and Reagents

Milli-Q water (H_2O)

AmBic (Ammonium Bicarbonate) solution: 40 mM NH_4HCO_3 in H_2O

ACN solution: 50% acetonitrile in 0.5% formic acid

Lysis buffer: 5% (w/v) sodium dodecyl sulfate (SDS), 50mM Tris/HCl pH 7.6

STrapping buffer: 90% methanol, 100 mM Tris/HCl pH 7.1

Phosphoric acid solution: 12.15% in H_2O

DTT solution: 1 M dithiothreitol in H_2O, freshly prepared

TCEP elution solution: 40 mM tris(2-carboxyethyl)phosphine in H_2O, freshly prepared

IAA solution: 40 mM iodoacetamide in AmBic, freshly prepared

Trypsin solution: 0.07 µg/µl of trypsin (V5111, Promega) in AmBic, prepared prior to starting the digest and kept on ice

FA solution: 0.5% formic acid in H_2O

Isopropanol solution: 40% isopropanol in H_2O

Methanol

1M NaOH

SAX elution solution: 2% formic acid in H_2O

C_{18} STAGE tips

SAX STAGE tips

Equipment

Bench-top centrifuge (for example MiniSpin, Eppendorf)

Probe sonicator (for example Soniprep 150, MSE)

Heating block suitable for handling microtubes (for example PHMT, Grant Bio)

Plastic syringe, 20 ml (for example 301031, BD) with a custom adapter to fit into 200 µl pipette tips

Vacuum concentrator (for example SpeedVac, Thermo)

Only Quartz C-STrap tip

MK360 quartz filter is modified with pyridyldithiol, i.e. MK360-50 mm filter is incubated with 10 ml of 2% (3-Aminopropyl)trimethoxysilane (APTMS) soluton in acetone for 2 hours and then washed several times with acetone. 7 mg of *N*-succinimidyl 3-(2-pyridyldithiol)propionate (SPDP) is dissolved in 0.5 ml of dimethyl sulfoxide (DMSO) and added into 9.5 ml of the phosphate buffered saline (PBS)/EDTA solution (pH 7.4, 15 mM EDTA). The aminopropyl-modified filter is incubated with the resultant SPDP solution for 6 hours (or overnight) at room temperature (RT), and then washed several times with the PBS/15 mM EDTA solution and air dried.
Using a 14 gauge blunt needle, the OQ C-STrap tip is constructed by inserting 12 plugs of the pyridyldithiol modified MK360 material into a 200 µl pipette tip - similarly to the original STrap tip protocol[1] however no underlying C_{18} membrane compartment is added in this case.
O-tube: A 1.5 mL microcentrifuge tube (72.690.001, Sarstedt) with an opening punctured in the tube's lid (alternatively, a pipette tip adapter for microcentrifuge tubes could be used).
The C-STrap tip and O-tube comprise the **Spin-unit**
Filter tips, 10 µl (TF-300-R-S, Axygen)

2. Methods

2.1 Cell lysis and reduction of cysteine residues

Cells are lysed in excess of the Lysis buffer (ca. 1:10 sample-to-Lysis buffer volume ratios) at room temperature. To shear the DNA, the lysate is briefly sonicated with a probe sonicator. DTT solution is added to the final concentration of 20 mM. The extract is heated up at 95°C for 5 min. The extract is clarified by centrifugation at ~12,000 x g for 10 min.

2.2 Preparation of the Trypsin solution

Trypsin solution is prepared prior to the step 2.3 and placed on ice.

2.3 Sample processing

1. Pre-heat the heating block to 47°C.
2. (S**ee Notes**) Add 120 µl of the STrapping buffer into the C-STrap tip onto the top of the quartz stack. Wait for 1 min.
3. (S**ee Notes**) To 18 µl of the sample add 2 µl of the Phosphoric acid stock solution. Mix by pipetting up and down.
4. Slowly add the acidified sample into the upper quarter of the STrapping buffer in the C-STrap tip. Insert the C-STrap tip into the O-tube. Place the Spin-unit into the centrifuge and mark the C-STrap tip part facing outwards.
5. Centrifuge the Spin-unit at 3000 x g for 1 min. Dispose of the tube with the flow-through.

6. Add 70 µl of the STrapping buffer into the C-STrap tip. Insert the tip into the fresh O-tube. Place the Spin-unit into the centrifuge with the C-STrap tip mark facing inwards. Centrifuge the Spin-unit for 30 sec at 3000 x g.

7. (Optional) Add 40 µl of the AmBic solution into the C-STrap tip and centrifuge the Spin-unit for 30 sec at 3000 x g. Dispose of the tube with the flow-through.

8. Add 30 µL of the Trypsin solution into the C-STrap tip onto the top of the plug stack. Push down the solution using the syringe with a customized tip adapter till the solution meniscus is positioned ca. 4 mm above the top of the plug stack.

9. Close the top of the C-STrap tip with the 10 µl filter tip.

10. Insert the closed C-STrap tip into the fresh O-tube, place the unit into the heating block at 47°C and cover with the aluminium foil. Incubate for 60 min.

11. Remove the Spin-unit from the heating device. Add 50 µl of the AmBic solution into the C-STrap tip.

12. Centrifuge the Spin-unit at 3000 x g for 30 sec. Collect the flow-through fraction containing unbound non-Cys peptides. The peptides in this fraction could be further fractionated by ion-exchange (e.g. by the SAX STAGE tip fractionation[3]).

13. The C-STrap tip is washed consecutively with 50 µl of AmBic solution, 70 µl of FA solution, 70 µl of the ACN solution, and, optionally, 40 µl of the isopropanol solution using the Spin-unit centrifugation and decanting the flow-through when necessary.

14. The C-STrap tip is washed with 40 µl of the AmBic solution.

15. 40 µl of the TCEP elution solution is added into the C-STrap tip. The tip is placed into a fresh O-tube, the elution solution is pushed down with the syringe-adapter till the solution meniscus is positioned ca. 5 mm above the top of the plug stack. Incubation is performed for 30 min at room temperature.

16. 30 µl of the AmBic solution is added into the C-STrap tip. The Spin-unit is centrifuged at 3000 x g for 30 sec.

17. The collected flow-through is loaded onto the C_{18} STAGE tip activated with the consecutive washes of methanol and the FA solution[3].

18. The STAGE tip is washed with 50 µl of the AmBic solution.

19. Add 40 µL of the IAA alkylation solution into the tip. Push down the solution using the syringe with a customized tip adapter till the solution meniscus is positioned ca. 5 mm above the top of the plug stack. Leave the tip at RT in the dark for 15 min.

20. Spin down the STAGE tip. Wash with the FA solution.

21. Elute the peptides with the ACN solution. Concentrate the eluted peptides in SpeedVac.

SAX STAGE tip fractionation into 2 fractions is performed using the [2] reference as a guideline with some modifications

The SAX tip is activated with the consecutive washes of methanol, 1M NaOH, and AmBic.

The non-Cys fraction is loaded onto the tip. The flow-through is collected (SAX1 fraction).

The tip is washed with AmBic. The elution is performed with the SAX elution solution (SAX2 fraction).

The SAX fractions are cleaned-up using the C_{18} STAGE tips.

Notes:

3. After each centrifugation step make sure that all added solution has gone through the tip.
4. The typical working ratio between the STrapping buffer in the C-STrap tip and the acidified sample is 6:1. The typical final concentration of the phosphoric acid after addition to the sample is 1.2%.

References:

1 Zougman, A., Selby, P. J. & Banks, R. E. Suspension trapping (STrap) sample preparation method for bottom-up proteomics analysis. *Proteomics* **14**, 1006-1000, doi:10.1002/pmic.201300553 (2014).

2 Wisniewski, J. R., Zougman, A. & Mann, M. Combination of FASP and StageTip-based fractionation allows in-depth analysis of the hippocampal membrane proteome. *J Proteome Res* **8**, 5674-5678, doi:10.1021/pr900748n (2009).

3 Rappsilber, J., Mann, M. & Ishihama, Y. Protocol for micro-purification, enrichment, pre-fractionation and storage of peptides for proteomics using StageTips. *Nature protocols* **2**, 1896-1906, doi:10.1038/nprot.2007.261 (2007).
